# Characterization of the Population, Treatment Patterns, and Outcomes of Patients with Advanced or Metastatic Non-Small-Cell Lung Cancer (NSCLC) with Epidermal Growth Factor Receptor Mutation (EGFRm): A Retrospective Cohort Study from IPO Porto

**DOI:** 10.3390/curroncol32080414

**Published:** 2025-07-24

**Authors:** Ana Rodrigues, Marta Pina, Rita Calisto, Pedro Leite-Silva, Pedro Medeiros, Catarina Silva, Ana Sofia Silva, Patrícia Redondo, João Ramalho-Carvalho, Susana Ferreira Santos, Maria José Bento

**Affiliations:** 1 Medical Oncology Department, Portuguese Oncology Institute of Porto, 4200-072 Porto, Portugal; 2 Outcomes Research Lab, Portuguese Oncology Institute of Porto, 4200-072 Porto, Portugal; 3 Group of Epidemiology, Results, Economy and Management in Oncology, GEREMO Research Center, Portuguese Oncology Institute of Porto, 4200-072 Porto, Portugal; 4 Porto Comprehensive Cancer Center (Porto.CCC) & RISE@CI-IPOP (Health Research Network), Portuguese Oncology Institute of Porto, 4200-072 Porto, Portugal; 5 Epidemiology Department, Portuguese Oncology Institute of Porto, 4200-072 Porto, Portugal; 6 Institute for Evidence Based Health (ISBE), 1649-028 Lisbon, Portugal; 7 J&J Innovative Medicine, 2740-298 Lisbon, Portugal

**Keywords:** real-world, retrospective, non-small-cell lung cancer (NSCLC), EGFR, mutation, treatment, outcomes, overall survival, progression-free survival

## Abstract

Most patients with non-small-cell lung cancer (NSCLC) present with advanced/metastatic disease at diagnosis, and molecular profiling is critical in guiding treatment decisions. This retrospective cohort study aimed to characterize EGFR mutations (EGFRm) in advanced/metastatic NSCLC patients, treatment patterns, and real-world outcomes. Adults diagnosed between 2018 and 2021 and treated at a Comprehensive Care Center were included. Time-to-event outcomes were analyzed using the Kaplan–Meier method. A total of 110 patients were included, with a median age of 69.0 years (range, 37–93), 76.4% female, and 83.2% non-smokers. About 97.3% had adenocarcinomas, with 93.6% at stage IV, 40.9% with ≥ three metastatic sites (brain metastases in 24.5%), 33.6% ECOG 2–4, and 58.2% with an EGFR exon-19 deletion. A minority started supportive care or curative-intent treatment, and 81.8% underwent first-line palliative systemic therapy (TKIs, 91.1%; chemotherapy, 8.9%). Median real-world overall survival (rwOS) was 18.9 months (95% CI, 13.8–28.1). Worse rwOS was observed in patients with ECOG 2–4 versus ECOG 0–1 (10.3 vs. 22.8 months; HR 1.82, 95% CI 1.17–2.85; *p* = 0.008) and in patients with exon-21 L858R versus exon 19 deletions (15.8 vs. 24.2 months; HR 1.59, 95% CI 1.00–2.54; *p* = 0.048). In patients treated with palliative systemic treatment, median progression-free survival was 10.9 months (95% CI, 8.8–13.6). This study provides important insights regarding real-world characteristics, treatment patterns, and outcomes from a cohort of EGFRm advanced/metastatic NSCLC patients.

## Simple Summary

This study looked at real-world data from patients with advanced or metastatic non-small-cell lung cancer who had a specific genetic change called an EGFR mutation. Although it is known that EGFR testing is important to guide treatment, real-life information on how these patients are treated and how they fare is limited. By reviewing records from a Portuguese cancer center between 2018 and 2021, this study found that most patients were older, female, and non-smokers, with many having advanced disease and multiple metastases. The majority received targeted therapy, and survival varied depending on their overall health and type of EGFR mutation. These findings offer valuable insights into how EGFR-mutated non-small-cell lung cancer is managed in everyday practice, highlighting differences in outcomes that could inform future research, enhance clinical guidelines, and support more personalized treatment decisions in real-world settings.

## 1. Introduction

Lung cancer is still one of the most complex global health challenges, being the most common cancer and the leading cause of cancer-related deaths according to the most recent worldwide statistics (2022) [1]. Lung cancer accounted for approximately 2.48 million new cases worldwide and had 1.8 million attributed deaths [1].

Non-small-cell lung cancer (NSCLC) is the most common type of lung cancer, accounting for approximately 85% of all primary lung cancers [2]. Patients are often diagnosed at an advanced or metastatic stage [2]. The 5-year overall survival rate for NSCLC may vary between 68% in stage IB and 0–10% in stage IVA–IVB disease [2]. For patients with epidermal growth factor receptor (EGFR) mutations, the estimated 5-year survival rate for EGFR exon 20 insertions is 8% compared with 19% for common EGFR mutations [3].

Over the past decade, improvements in the molecular profiling of NSCLC have led to a more profound understanding of the oncogenic drivers, with EGFR mutations emerging as one of the most important actionable targets for the use of precision therapy [4]. These mutations occur in approximately 15% of NSCLC cases in Caucasian populations and can reach up to 50% in Asian populations [5]. The most common alterations include exon 19 deletions (45% of all EGFR-activating mutations) and the L858R point mutation in exon 21 (40–45%), which together comprise most EGFR-driven NSCLC cases [6].

With the development of targeted therapies in oncology, particularly tyrosine kinase inhibitors (TKIs), the treatment landscape for EGFR-mutated patients was revolutionized. In particular, third-generation TKIs transformed the standard frontline treatment for advanced EGFR-mutant NSCLC as the preferred first-line treatment in clinical guidelines [7], owing to better efficacy and fewer side effects [8]. Nevertheless, their effectiveness seems to have reached a plateau, as the median progression-free survival (PFS) duration in significant trials has not come close to two years for any approved third-generation EGFR TKI as a monotherapy [9]. Moreover, the extent of benefits from these treatments varies significantly among different EGFR mutations (i.e., Del 19 vs. L858R). Although new combinations enhance both the PFS and duration of response, the specific type of common EGFR mutation still has an impact on the degree of benefit [10,11]. There is a subset of mutations, particularly exon 20 insertions, which can occur in around 4–15% of all EGFR-mutated cases, where therapeutic advances have historically been limited and correlated with worse clinical outcomes [12]. Moreover, even common EGFR mutations show a high level of heterogeneity, as patients with the EGFR exon 19 deletion exhibited PFS, OS, and higher response rates following EGFR-TKI therapy compared to those with the exon 21 L858R mutation [13]. As research continues to elucidate the biologic complexity of EGFR-mutant NSCLC, the development of strategies aimed at extending clinical benefits remains a priority. Investigational approaches include combining third-generation EGFR TKIs with anti-angiogenic agents and chemotherapy, utilizing the EGFR–MET bispecific antibody amivantamab, and applying local ablation strategies for oligoprogressive disease. These are being explored to delay acquired resistance, extend the duration of response, and enhance treatment efficacy [14].

With this increasing complexity, comprehensive genomic profiling has become pivotal in guiding treatment decisions. Next-generation sequencing (NGS) emerged as the gold standard for molecular testing, allowing for the identification of both common and rare EGFR alterations, while it has facilitated the identification of new molecular markers that may serve as prognostic and predictive biomarkers, emphasizing the importance of personalized medicine in guiding treatment choices and enhancing patient outcomes.

Despite this, accessibility to these testing methods remains a challenge, which can lead to underdiagnosis and suboptimal treatment selection in many cases [15]. A recent retrospective analysis of electronic health records from the US demonstrated significant gaps in EGFR testing and its impact on treatment selection and patient outcomes [16]. There is a significant proportion of patients with EGFR exon 20 insertions and uncommon EGFR mutations who remain undiagnosed due to suboptimal diagnostic methods [17], and these subgroups have attracted growing attention, primarily due to the increasing use of NGS in clinical practice. Addressing this unmet need is essential to ensure that all eligible patients receive the most appropriate and effective therapies.

Therefore, the aim of this study was to evaluate the treatment patterns and outcomes in patients with EGFR-mutated advanced or metastatic NSCLC, diagnosed between 2018 and 2021 and treated in a Portuguese Comprehensive Cancer Center (IPO Porto). The main goal of this study was to outline the demographic and clinical characteristics of patients, based on the mutational profiling of EGFR (common mutations such as exon 19 deletions and L858R mutations and uncommon EGFR mutations). Secondary goals included assessing treatment patterns and real-world clinical outcomes such as overall survival, progression-free survival, time to central nervous system (CNS) metastasis, time to next treatment, and time on treatment. By providing a comprehensive analysis of EGFRm NSCLC, this study seeks to enhance clinical decision-making and contribute to optimizing patients’ outcomes.

## 2. Materials and Methods

### 2.1. Study Design

LuCaRE was an observational, retrospective, cohort, single-center, secondary-use data study including patients with EGFRm advanced or metastatic NSCLC diagnosed between 1 January 2018 and 31 December 2021 at IPO Porto (a Comprehensive Cancer Center), who were followed up with in an Outpatient Clinic of Medical Oncology.

For each patient, data were retrospectively retrieved from the date of NSCLC diagnosis (index date) until the earliest of the patient’s last contact, death, or data cut-off (31 December 2023), spanning a maximum follow-up period of 5 years. The study database was locked in March 2024, and the final study report was released in April 2025.

This study was approved by the Board of Administration and the Ethics Committee of IPO Porto, and by the Data Protection Officer before data collection. Patients’ informed consent was exempt from the Ethics Committee of IPO Porto due to the retrospective observational nature of this study and data anonymization. The reporting of this study conforms to the Strengthening the Reporting of Observational Studies in Epidemiology (STROBE) statement [18].

### 2.2. Selection of Patients

Eligible patients were male or female, aged 18 years or older, with histologically or cytologically confirmed advanced or metastatic NSCLC (TNM stage IIIb, IIIc, or IV [19]) diagnosed between January 2018 and December 2021, positive for EGFR mutations, and followed-up with at one of the outpatient clinics of Medical Oncology at IPO Porto. Patients were excluded if they had received NSCLC treatment outside IPO Porto at any time, did not have follow-up by a multidisciplinary team or medical oncology, had another malignancy within the past five years or during the study (except for cutaneous basal cell or squamous cell carcinoma), or were enrolled in a clinical trial.

### 2.3. Data Collection

Demographic, clinical, and treatment data were extracted from electronic medical and administrative records at IPO Porto. The retrieved data underwent thorough quality control procedures to ensure integrity and accuracy. Confidentiality and anonymization of the data for analysis were strictly maintained. Data linkage between sources was accomplished using the unique internal identifier assigned by IPO Porto.

### 2.4. Outcomes

Real-world outcomes were computed from the extracted data by utilizing the following definitions:

- Real-world overall survival (rwOS): Time from diagnosis to death from any cause (if the event did not occur, time was censored at data cut-off as death was fully traced);
- Real-world progression-free survival (rwPFS): Time from the start of the first line of therapy (LoT) and first documented disease progression or death from any cause, whichever occurred first (if an event was not identified, time was censored at last contact);
- Real-world time to CNS metastasis: Time between the start of LoT1 and the first detection of a new CNS metastasis (if an event was not identified, time was censored at last contact);
- Real-world time to next treatment (rwTTNT): Time between the start of LoT1 and the initiation of LoT2 (if an event was not identified, time was censored at last contact or death, whichever occurred first);
- Real-world time on treatment (rwToT): Time between the start of LoT1 and the date of the last administered drug of whichever LoT or death, whichever occurred first (if an event was not identified, time was censored at last contact); time during treatment interruptions between LoTs was included in the calculation of rwToT.

### 2.5. Statistical Analysis

All patients at IPO Porto fulfilling the eligibility criteria were included in the statistical analysis (no formal sample size calculation was performed). The statistical analysis was conducted for the overall sample and stratified by EGFR mutation category (common/uncommon) and type of mutation, whenever possible. Demographic, clinical, and treatment characteristics were summarized using descriptive statistics. The treatment pattern was described as the percentage of patients by drug and drug class at each line of treatment (LoT) with the corresponding median time in LoT. Transitions between treatments over LoTs were displayed using Sankey diagrams. Time-to-event outcomes were analyzed using the Kaplan–Meier methodology and reported as the median, rates at landmarks, and corresponding 95% confidence intervals (CIs). The log-rank test was used to compare survival curves between subgroups, together with hazard ratios from non-adjusted Cox regressions.

This analysis was performed by considering only valid observations. No imputation method was carried out for missing data. A *p*-value less than 0.05 was considered statistically significant. The statistical analysis was performed using Microsoft Office Excel 2019 version 1808 and R software version 4.0.5.

To comply with data protection regulations and institutional policy, all patient counts below six (*n* ≤ 5) were suppressed. Secondary suppression was also applied in cases where masked values could be inferred by subtraction.

## 3. Results

From a total of 3389 screened patients diagnosed with NSCLC between 2018 and 2021, 110 were included and 3279 were excluded: 1259 were EGFR-negative, 813 had early-stage NSCLC, 689 were treated outside of IPO at any time, 493 had co-malignancies, and 25 were enrolled in clinical trials (more than one criterion may have been fulfilled). The included patients were evenly distributed across the index year (20 patients in 2018 and 30 patients in each following year). The median follow-up was 18.9 months (range: 0.5–64.0 months).

### 3.1. EGFR Mutations

Approximately 90% of patients (*n* = 99) harbored common EGFR mutations, while 10% (*n* = 11) exhibited uncommon mutations. Among common mutations, exon 19 deletions were the most prevalent, observed in 58.2% of cases, followed by exon 21 L858R mutations, which accounted for 31.8% (Table 1). NGS was the predominant method utilized for molecular profiling, employed in 72.7% of cases, while PCR was utilized in 27.3% of cases.

### 3.2. Demographic and Clinical Characteristics at Diagnosis

At the time of initial NSCLC diagnosis, patients’ ages ranged from 37 to 93 years, with a median age of 69 years. Most were female (76.4%), and 83.2% had never smoked. Most patients had an ECOG PS of 0–1 (66.4%), whereas about one-third (33.6%) had an ECOG PS of 2–4 and stage IV disease (93.6%). The histological type of NSCLC was predominantly adenocarcinoma (97.3%). At diagnosis, 58.2% had 1–2 metastasis sites, 40.9% had three or more metastasis sites, and only one patient had no metastasis. About 24.5% and 5.5% of patients had brain or liver metastases, respectively (Table 1).

### 3.3. Characteristics at Disease Progression

Metastasis status, weight, and ECOG at disease progression are described in the Supplementary Materials (Tables S1–S3).

### 3.4. Frontline Treatment Intention

**Figure 1:**
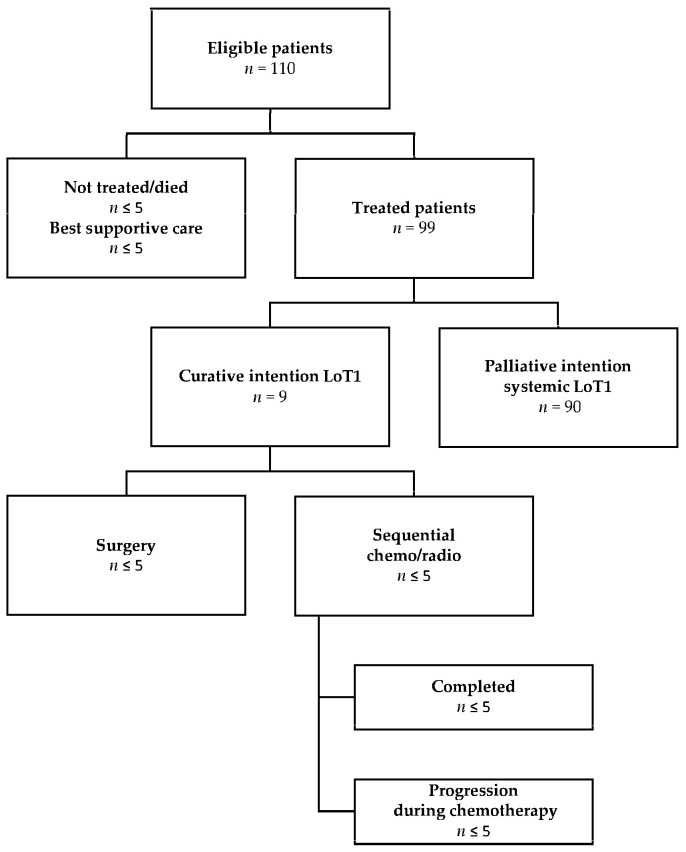
Patient flowchart by chosen treatment after diagnosis.

Out of the 110 included patients, fewer than 9.0% commenced best supportive care (in some instances following antalgic and/or whole-brain radiotherapy), about 82.0% initiated systemic palliative treatment, fewer than 9.0% started curative intention treatment (in stage III or stage IV oligometastatic disease), and fewer than five patients died shortly after diagnosis and without receiving any treatment (Figure 1).

Among patients initiating LoT1 curative intention treatment (*n* = 9), a small group underwent either sequential chemoradiotherapy or surgery (in some cases after performing neoadjuvant chemotherapy). Given that all nine patients experienced disease progression, some initiated palliative treatment with a TKI, whereas patients who carried an exon 20 insertion and PD-L1 expression higher than 50% started immunotherapy.

Of the patients presenting with brain metastases at the time of diagnosis, nine (33.3%) underwent whole-brain radiotherapy.

### 3.5. Treatment Dynamics

Table 2 summarizes treatments over LoT in patients who started palliative systemic treatment (*n* = 90). At data cut-off, 46.7% (*n* = 42) of these patients had progressed to LoT2, 20% (*n* = 18) to LoT3, less than 6% reached LoT4, and fewer achieved LoT5.

The treatment most frequently administered to patients in LoT1 was a TKI, which accounted for 91.1% of cases. Nearly half of the patients (48.9%) received a first-generation TKI, while 24.4% were treated with a third-generation TKI, and 17.8% with a second-generation TKI. The remaining patients (*n* = 8, 8.9%) received chemotherapy in LoT1 (all before having EGFR results available). Of the 48 patients who were not in LoT2, 13 were still on LoT1 at data cut-off, whereas 35 had their treatment suspended. Among those suspended, a small number of patients experienced clinical deterioration (LoT1 performed for less than 60 days), 27 had clinical deterioration and were deemed unfit for LoT2, a few patients were proposed for LoT2 chemotherapy but died, and some died outside of the institution without a clear indication of disease progression at last contact.

In LoT2, TKI continued to be the most common treatment (61.9%), while the percentage of patients receiving chemotherapy increased to 35.7%. Patients with clinical conditions were treated with chemotherapy or osimertinib if not used in LoT1. Among the 21 patients treated with osimertinib in LoT2, 17 harbored the EGFR T790M mutation as the mechanism of resistance. In seven of these patients, the mutation was identified through a tumor biopsy following a lack of identifiable mutations in the liquid biopsy test.

In LoT3, one-third of patients were treated with TKI (33.3%), and 66.7% were treated with chemotherapy. In LoT4, a small number of patients received chemotherapy (docetaxel or vinorelbine), and some patients were given docetaxel in combination with nintedanib or treated with a TKI (afatinib). In LoT5, a very small number of patients were treated with a TKI (erlotinib) as a rechallenge.

**Figure 2:**
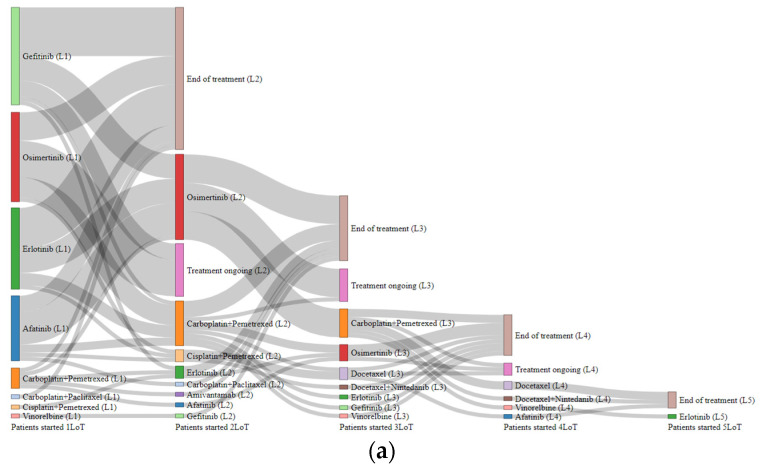

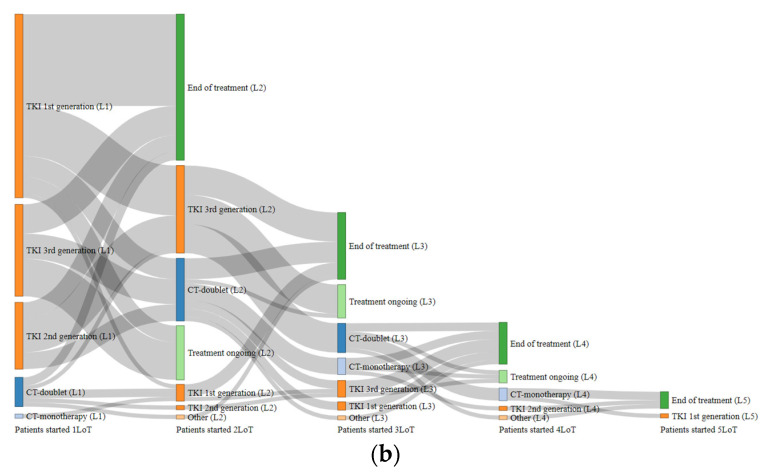
Sankey plot for the patient’s journey (**a**) by drug and (**b**) drug class in patients who initiated LoT1 with systemic palliative treatment (*n* = 90). Each drug/drug class is represented as a column with a unique color. The size of each column is proportional to the number of patients receiving that drug/drug class in each LoT. Gray paths represent switching pathways between LoTs. End of treatment includes deaths or switches to BSC. Abbreviations: first-generation TKI: erlotinib and gefitinib; second-generation TKI: afatinib; third-generation TKI: osimertinib; CT-monotherapy: docetaxel and vinorelbine; CT-doublet: carboplatin + vinorelbine, carboplatin + paclitaxel, cisplatin + pemetrexed, and carboplatin + pemetrexed; Other: docetaxel + nondecane and amivantamab.

The treatment journey is depicted by drug and drug class in Figure 2.

Regarding patients who started treatment with a curative intention (*n* = 9), some patients underwent surgery, with fewer after neoadjuvant chemotherapy. In a small number of patients, systemic palliative treatment was initiated immediately after surgery due to rapid progression after resection or incomplete resection. The remaining patients were recommended sequential chemoradiotherapy: a low number of patients experienced disease progression during chemotherapy and could not undergo radiotherapy; some patients completed the planned chemoradiotherapy but progressed 6–9 months after the end of treatment.

### 3.6. Real-World Outcomes

**Figure 3:**
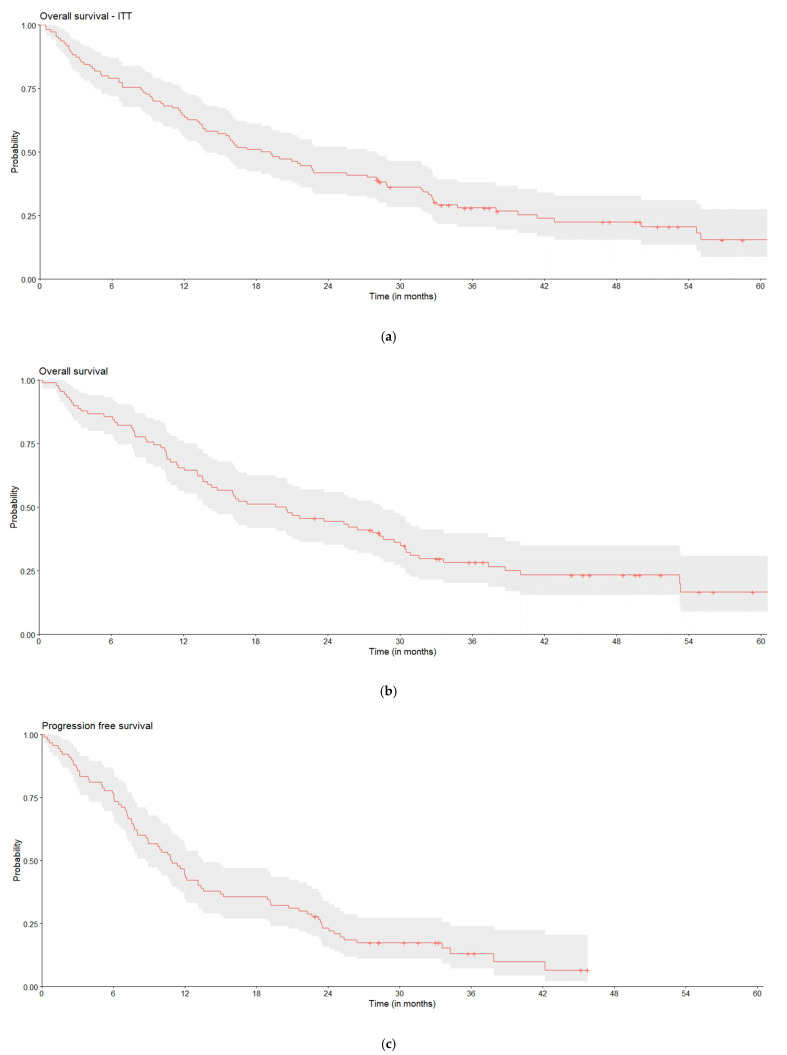

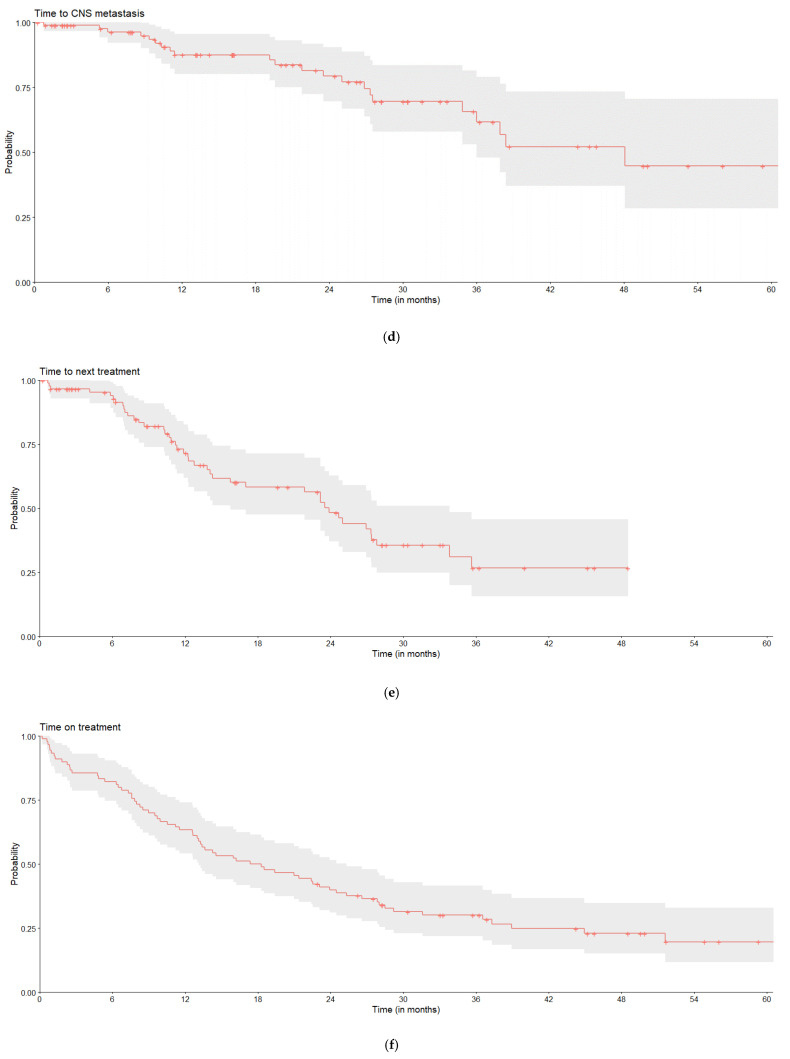
Real-world clinical outcomes, including the overall (**a**) rwOS (*n* = 110), for patients who initiated LoT1 with systemic palliative treatment. (**b**) rwOS (*n* = 90), (**c**) rwPFS (*n* = 90), (**d**) time to CNS metastasis (*n* = 90), (**e**) rwTTNT (*n* = 90), and (**f**) rwToT (*n* = 90).

Real-world outcomes are presented in Table 3 and Figure 3. Except rwOS, all other outcomes were calculated only for the subgroup of patients who started palliative systemic treatment in LoT1 as the number of patients starting curative intention treatment was very low.

Overall, 85 (77.3%) patients died during the study period. The median rwOS was 18.9 months (95% CI: 13.8–28.1), and survival rates were 64.5% in year 1, 41.8% in year 2, 28.0% in year 3, and 15.4% in year 5.

Among patients treated with systemic palliative treatment in LoT1 (*n* = 90), the median rwOS was 21.1 months (95% CI: 16.0–31.8). At data cut-off, 86.7% of these patients had confirmed disease progression, and the median rwPFS was 10.9 months (95% CI: 8.8–13.6).

Within this same subgroup, twenty-two patients (22.4%) developed new CNS metastases, comprising fifteen patients who experienced their first CNS metastasis and seven patients who developed additional CNS metastases. The median time to CNS metastasis was 48.1 months (95% CI: 36.0–NA). The median rwTTNT was 23.9 months (95% CI: 15.7–33.8), while the median rwToT was 17.8 months (95% CI: 13.1–25.3).

**Figure 4:**
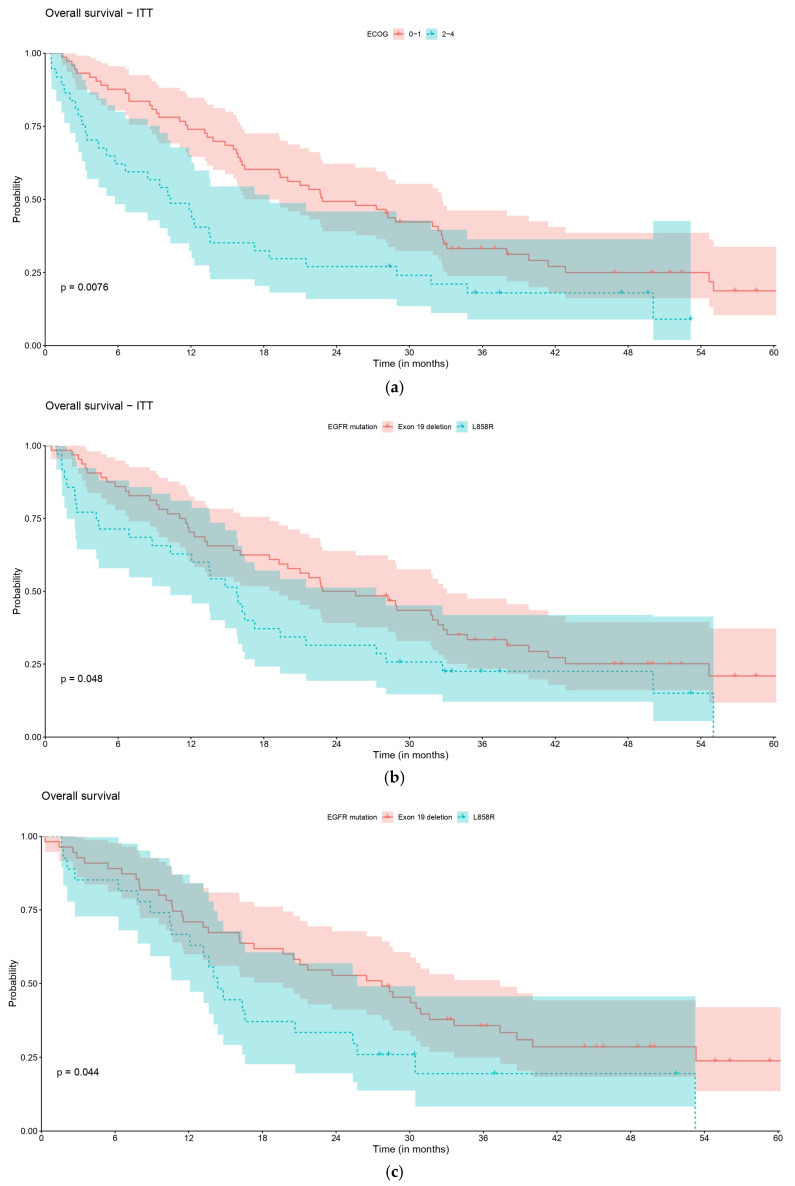

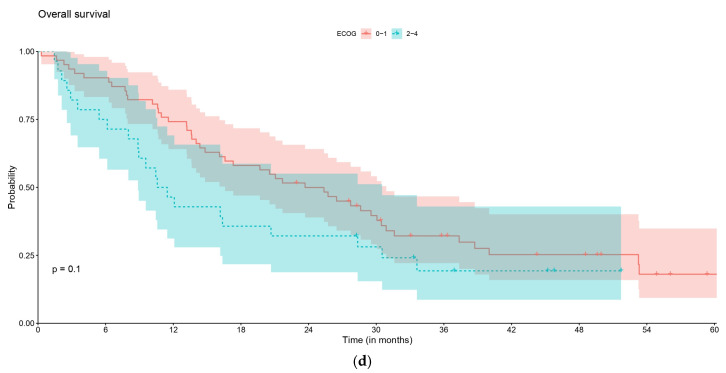
Comparison of real-world OS: (**a**) rwOS by ECOG (*n* = 110) and (**b**) rwOS by mutation (*n* = 99) in the overall population (*n* = 110); (**c**) rwOS by ECOG (*n* = 90) and (**d**) rwOS by mutation (*n* = 82) in the subgroup of patients who initiated LoT1 with systemic palliative treatment (*n* = 90). Other comparisons are available in the Supplementary Materials (Figures S1–S3, Tables S4 and S5).

Statistical comparisons of survival curves between subgroups are presented in Table 4 and Figure 4. ECOG and EGFR mutation significantly impacted rwOS. rwOS was significantly lower in patients with ECOG PS 2–4 compared to patients with ECOG PS 0–1 [10.3 months (95% CI, 5.8–18.5) vs. 22.8 months (95% CI: 19.3–32.7), HR 1.82 (95% CI, 1.17–2.85), *p* = 0.008], as well as in patients with an Exon 21 L858R mutation compared to those with an EGFR Exon 19 deletion [15.8 months (95% CI: 10.3–27.3) vs. 24.2 months (95% CI, 19.3–33.1), HR 1.59 (95% CI, 1.00–2.54), *p* = 0.048]. No statistically significant differences were depicted in other variables. Other analyses are available in the Supplementary Materials (Figures S1–S3, Tables S4 and S5).

## 4. Discussion

The LuCaRE study investigated real-world treatment patterns and clinical outcomes in a cohort of 110 patients diagnosed with advanced-stage NSCLC and EGFR mutations at a tertiary comprehensive institute. Earlier studies have demonstrated that real-world data can offer valuable comparisons to clinical trial data concerning outcome measures in NSCLC, such as rwPFS, rwOS, and rwORR [20,21].

According to the literature, approximately 85–90% of EGFR mutations comprise Exon 19 deletions and L858R point mutations (common EGFR mutations), while the remaining 10–15% comprise uncommon mutations [22]. Our study reported comparable findings (90% common and 10% uncommon EGFR mutations) despite the small sample size. EGFR exon 20 insertion mutations are the third most common type of EGFR mutation in NSCLC [22], as observed in our study, and are estimated to account for 0.1% to 4.0% of all NSCLC cases [23] (0.2% at IPO Porto, with six out of 3389 cases) and 4% to 12% of all EGFR mutations [23] (5.5% at IPO Porto, with six out of 110 cases). However, exon 20 insertions may be underrepresented in the LuCaRE study, potentially due to the use of PCR in a significant proportion of patients (27.3%). It has been shown that PCR tests miss more than 40% of patients with NSCLC harboring EGFR exon 20 insertion mutations. NGS-based genetic testing is preferable to standard PCR assays and can substantially improve the identification of the diverse profile of EGFR exon 20 insertion variants in NSCLC, as well as other atypical EGFR mutations [24].

Real-world studies on the treatment and survival of EGFR-mutated NSCLC have their advantages and disadvantages [25]. The data were collected from a single center and included 17.3% octogenarians and 33.6% of patients with an ECOG PS ≥ 2, which are typically excluded from clinical trials. Additionally, 40.9% of the patients presented with ≥3 metastatic sites, suggesting a high tumor burden. Moreover, brain metastases were diagnosed in 24.5% of patients, and the real prevalence could be even higher if a brain MRI is conducted before treatment initiation, such as in the recent MARIPOSA [10] and FLAURA-2 [11] trials, which reported a rate of around 40%. Our estimate corresponds to the lower limit of the 95% confidence interval for the proportion of advanced/metastatic NSCLC patients with brain metastasis found in a recent systematic review (29.4%; 95% CI, 24.5–34.5) [26].

Studies have shown that EGFR mutations in NSCLC are more common in women, light or non-smokers, patients with adenocarcinoma histology, and Asian populations [23]. The characteristics of the patients included in LuCaRE are consistent with these findings, even though ethnicity was not assessed in this study. Demographic characteristics appear comparable between the subgroup of patients with common mutations and those with uncommon mutations. However, this finding should be viewed with caution due to the small number of patients with uncommon mutations. The same limitation applies to the comparison of disease volume at diagnosis between these two subgroups.

The comprehensive data on treatment patterns allowed an in-depth understanding of the way patients with EGFRm NSCLC were managed at IPO Porto between 2018 and 2023. This analysis revealed that treatment strategies for real-world patients with EGFR-mutated NSCLC are heterogeneous, reflecting, at that time, the use of different TKIs in front-line therapy but also the complexity of second-line treatments, for which patients carrying the T790M mutation were treated with osimertinib.

In the LuCARE study, a limited cohort of patients underwent treatment with curative intent for stage IIIb or stage IV oligometastatic disease, which included radical interventions such as surgical resection or chemotherapy/radiotherapy. Conversely, most patients commenced systemic treatment, primarily utilizing TKIs (mainly first-generation TKIs) as the main therapeutic modality. For those with conditions that suggested second-line treatment, the option was between osimertinib, if the T790M mutation was identified as a mechanism of resistance, or chemotherapy for the remaining patients. Indeed, osimertinib as a first-line treatment was used in a small portion of patients. To accurately interpret these findings, it is crucial to consider the availability and use of first- and second-line treatments. Osimertinib was officially reimbursed as a second-line treatment for patients with the EGFR T790M mutation on 25 January 2018, although it may have been accessible earlier through clinical trials or early-access programs. As a first-line treatment, osimertinib became available through an expanded access program in 2019 and was reimbursed in Portugal on 13 March 2021. The study dates reflect this period and approval milestones.

A comprehensive study [16] conducted in the USA, from 2015 to 2021, revealed that among advanced/metastatic EGFRm NSCLC patients receiving a first-line treatment, 70.6% were treated with a TKI, 13.9% with chemotherapy, 10% with immunotherapy, and 5.4% with other treatments. Another study [27], including patients with metastatic EGFRm NSCLC receiving first-line systemic therapy between 2018 and 2021, found that 84% were treated with TKIs, 14% with chemotherapy, 0.9% with chemo-immunotherapy, and 0.7% with immunotherapy. Our results are generally aligned with these studies, except for immunotherapy, which, in fact, is not recommended for EGFRm NSCLC.

Our real-world cohort had a median OS of 18.9 months (95% CI: 13.8–28.1) and a median PFS of 10.9 months (95% CI: 8.8–13.6). Burnett H et al. (2021) [22] conducted a systematic review and reported a median OS ranging between 19.6–27.7 months and a median PFS between 8.5–15.2 months in patients with common EGFR mutations treated with a TKI. Stalker M et al. (2024) [27] reported a median OS of 35.4 months (95% CI, 32.7–37.9) for patients receiving first-line systemic therapy, which is significantly higher than our median OS in patients who started LoT1 (21.2 months). Consistent with the LuCaRE study, the authors reported a median OS of 38.7 months (95% CI: 35.9–46.3) for patients with EGFR exon 19 deletions compared to 33.9 months (95% CI: 27.9–38.0) for those with L858R mutations, thus confirming the previously described superior OS for patients with EGFR del19 versus L858R mutations [13]. These findings are further supported by a Dutch study that included 57,592 patients from 1 January 2015 to 31 December 2020. In this cohort, 1109 patients were identified, with 654 (59%) having del19 mutations and 455 (41%) with L858R mutations. The median OS was significantly better for patients with del19 mutations compared to those with L858R mutations (28.4 months vs. 17.7 months, *p* < 0.001), in line with LuCaRE data. A multivariable analysis showed no differences in survival among the various TKIs for both mutation groups, except in the subgroup of del19 patients with baseline BM, where osimertinib demonstrated a survival benefit [28].

Importantly, real-world data systematically indicated significantly poorer outcomes in treatment duration and effectiveness compared to the results from landmark clinical trials. This discrepancy may be linked to patients’ characteristics, such as ECOG status; however, the small sample size in our series restricts the clinical implications of these findings.

The LuCaRE study is subject to several limitations inherent to its reliance on secondary data sourced from the IPO Porto clinical registry, which constrains its findings due to the retrospective nature of the analysis. Such limitations include the potential for incomplete patient records, restrictions on the available registered data, and gaps in the documentation of treatment sequences and outcomes, as well as the risk of misclassification and under-reporting of critical data. Additionally, as a national single-center study, the results may lack representativeness for other Portuguese centers and may not be generalizable to diverse global populations. Furthermore, the therapeutic landscape for EGFR-mutated NSCLC in Portugal has evolved since the study period of 2018–2023. The inability to evaluate TP53 and other co-mutations due to the NGS panel used during the enrollment period, combined with the limited sample size for comparing outcomes in patients with rare mutations, is an additional study design limitation, even though co-mutations still have limited clinical implications. However, they are biologically relevant in order to understand the biology of the disease. It is worth noting that patient assessments (e.g., disease progression) in real-world practice often differ from those in clinical trials, potentially affecting outcomes such as PFS or time to CNS metastases. According to IPO guidelines, disease progression is evaluated at least at 3-month intervals using CT scans. Lastly, the inclusion of the objective response rate as a study outcome was impeded by outdated baseline evaluations, issues in comparing PET-CT and CT scans, and the inconsistent application of RECIST criteria in imaging assessments. Collectively, these factors underscore the necessity for cautious interpretation of the findings and their applicability to broader clinical contexts.

In conclusion, heterogeneity in study design, follow-up duration, data sources, treatment practices, and outcome definitions make non-adjusted comparisons between LuCaRE and other real-world studies challenging. Factors that might have negatively impacted our outcomes could have been the proportion of patients with ECOG ≥2, the number of metastatic sites, and other factors not captured by the present study, such as the socioeconomic context of the patient, lack of adequate support by caregivers and primary medical care, delay between admission at the institution and procedures of diagnosis, and staging and prescription of treatment. Another issue is the limited human medical resources at the institute, which restrict the personalized follow-up of patients with regular appointments, which is important to evaluate toxicities and for symptom management. Finally, a highlight about the overlap of the recruitment period and the COVID-19 pandemic. During the pandemic, there was a delay in seeking medical care by patients, as well as access constraints to hospitals and to workup diagnoses. In our results, this is perceived by the impossibility of performing NGS for all patients due to the lack of supply of materials. Genetic characterization by PCR might have had an impact on the number of insExon20 patients, for example.

## 5. Conclusions

The LUCARE study characterized EGFRm NSCLC patients, treatment patterns, and survival outcomes in a real-world setting. The real-world effectiveness of NSCLC treatments in LuCaRE is in line with what was previously observed in clinical trials. The heterogeneity across studies regarding study design, follow-up, data sources, treatment practices, and variability in outcome definitions complicates the indirect comparison between the LuCaRE study and other real-world studies. Factors that may have negatively influenced our outcomes include the proportion of patients with an ECOG performance status of ≥2, the number of metastatic sites, and other unmeasured variables such as the socioeconomic context of patients, inadequate care support, delays in diagnosis, staging, and treatment prescription processes. Additionally, the limited human medical resources at the institution hindered personalized follow-up, which is crucial for monitoring toxicities and managing symptoms. Furthermore, the overlap of the recruitment period with the COVID-19 pandemic introduced additional challenges; delays in patients seeking medical care and access to hospitals adversely affected diagnostic procedures. This is reflected in our results, as not all patients could undergo NGS due to material supply shortages. The reliance on PCR for genetic characterization may have influenced the number of patients identified with exon 20 insertions, for instance.

To our knowledge, this is the largest studied cohort of Portuguese EGFRm NSCLC patients. The study findings reflect routine practice in EGFR-mutated NSCLC and underscore real-world outcome differences that can guide future research, refine clinical guidelines, and support more personalized treatment decisions in a real-world setting.

## Figures and Tables

**Table 1:** Demographic and clinical characteristics at EGFRm NSCLC diagnosis, overall and by EGFR mutation category.

Characteristics	Overall (*n* = 110)	Common EGFR Mutation (*n* = 99)	Uncommon EGFR Mutation (*n* = 11)
**Median age (range), *years***	69.0 (37–93)	70.0 (37–93)	68.0 (46–81)
**Female, *n* (%)**	84 (76.4%)	75 (75.8%)	9 (81.8%)
**Never smoked, *n* (%)**	89 (83.2%)	79 (81.4%)	10 (100.0%)
**Weight < 80 kg, *n* (%)**	92 (86.8%)	84 (88.4%)	8 (72.7%)
**ECOG PS, *n* (%)**			
0	Not shown	26 (26.3%)	≤5
1	Not shown	40 (40.4%)	≤5
2	Not shown	21 (21.2%)	≤5
3–4	12 (10.9%)	12 (12.1%)	0 (0.0%)
**Adenocarcinoma, *n* (%)**	107 (99.1%)	97 (100.0%)	10 (90.9%)
**Unilateral, *n* (%)**	107 (100.0%)	96 (100.0%)	11 (100.0%)
**TNM stage, *n* (%)**			
IIIB-IIIC	Not shown	6 (6.1%)	≤5
IVA	30 (27.3%)	24 (24.2%)	6 (54.5%)
IVB	Not shown	64 (64.6%)	≤5
IV not specified	6 (5.5%)	≤5	≤5
**No. of metastasis sites, *n* (%)**			
0–2	65 (59.1%)	58 (58.6%)	7 (63.6%)
3–4	Not shown	32 (32.3%)	≤5
5–7	Not shown	9 (9.1%)	≤5
**Brain metastasis, *n* (%)**	Not shown	26 (26.3%)	≤5
**Liver metastasis, *n* (%)**	Not shown	19 (19.2%)	≤5
**Brain and liver metastasis, *n* (%)**	6 (5.5%)	6 (6.1%)	0 (0.0%)
**EGFR test type, *n* (%)**			
NGS	Not shown	70 (70.7%)	≤5
PCR	Not shown	29 (29.3%)	≤5
**EGFR mutation, *n* (%)**			
Exon 19 deletion	64 (58.2%)	64 (64.6%)	0 (0.0%)
L858R point mutation of Exon 21	35 (31.8%)	35 (35.4%)	0 (0.0%)
Exon 20 insertion	Not shown	0 (0.0%)	Not shown
Other mutation ^1^	Not shown	0 (0.0%)	Not shown

^1^ Other Exon 21, co-mutation T790M and Exon 19 deletion, co-mutation T790M and Exon 21 mutated, Exon 18 mutated, Exon 20-point mutation. Note: Percentages were calculated considering the total in each column (*n*) as the denominator; some counts are not shown to protect patient confidentiality due to small numbers.

**Table 2:** Treatments by LoT in patient who initiated LoT1 palliative systemic treatment (*n* = 90).

	LoT1 ^1^ (*n* = 90)	Median Time (Months)	LoT2 (*n* = 42)	Median Time (Months)	LoT3 (*n* = 18)	Median Time (Months)	LoT4 (*n* ≤ 5)	LoT5 (*n* ≤ 5)
**Drug, *n* (%)**								
Afatinib	16 (17.8%)	11.5	≤5	18.0	0 (0.0%)	NA	≤5	0 (0.0%)
Amivantamab	0 (0.0%)	NA	≤5	14.8	0 (0.0%)	NA	0 (0.0%)	0 (0.0%)
Carboplatin + paclitaxel	≤5	7.9	≤5	0.03	0 (0.0%)	NA	0 (0.0%)	0 (0.0%)
Carboplatin + pemetrexed	≤5	4.8	11 (26.2%)	3.7	0 (0.0%)	NA	0 (0.0%)	0 (0.0%)
Cisplatin + pemetrexed	≤5	7.6	≤5	2.8	7 (38.9%)	2.7	0 (0.0%)	0 (0.0%)
Docetaxel	0 (0.0%)	NA	0 (0.0%)	NA	≤5	1.7	≤5	0 (0.0%)
Docetaxel + nintedanib	0 (0.0%)	NA	0 (0.0%)	NA	≤5	0.03	≤5	0 (0.0%)
Erlotinib	20 (22.2%)	10.8	≤5	5.4	≤5	1.5	0 (0.0%)	≤5
Gefitinib	24 (26.7%)	9.6	≤5	0.5	≤5	1.2	0 (0.0%)	0 (0.0%)
Osimertinib	22 (24.4%)	6.2	21 (50.0%)	7.5	≤5	1.2	0 (0.0%)	0 (0.0%)
Vinorelbine	≤5	0.03	0 (0.0%)	NA	≤5	1.0	≤5	0 (0.0%)
**Drug class, *n* (%)**								
TKI 1st generation	44 (48.9%)	10.3	≤5	5.4	≤5	1.4	0 (0.0%)	n ≤ 5
TKI 2nd generation	16 (17.8%)	11.5	≤5	18.0	0 (0.0%)	NA	≤5	0 (0.0%)
TKI 3rd generation	22 (24.4%)	6.2	21 (50.0%)	7.5	≤5	1.2	0 (0.0%)	0 (0.0%)
CT-doublet	7 (7.8%)	7.6	15 (35.7%)	3.0	7 (38.9%)	2.7	0 (0.0%)	0 (0.0%)
CT-mono	≤5	0.03	0 (0.0%)	NA	≤5	1.0	≤5	0 (0.0%)
Other	0 (0.0%)	NA	≤5	14.8	≤5	0.03	≤5	0 (0.0%)

Notes: Percentages calculated for the number of patients in each LoT; time in each LoT was defined as the time between the first and last drug time of administration within each LoT (in patients who either discontinued treatment or transitioned to LoT2). This was only summarized until LoT3 due to the small sample size for LoT ≥ 4. ^1^ Eight patients had treatment modifications within LoT1 (patients underwent 1–2 cycles of chemotherapy due to severe symptoms while waiting for EGFR test results; however, once the results were available, the patients were switched to TKIs; patients experienced grade 3 toxicity to the TKI and were switched to an alternative TKI) and were not considered as starting a new LoT (longest treatment was considered as the LoT1). Abbreviations: NA: not applicable; first-generation TKI: erlotinib and gefitinib; second-generation TKI: afatinib; third-generation TKI: osimertinib; CT-mono: docetaxel and vinorelbine; CT-doublet: carboplatin + vinorelbine, carboplatin + paclitaxel, cisplatin + pemetrexed, and carboplatin + pemetrexed; Other: docetaxel + nintedanib and amivantamab.

**Table 3:** Real-world outcomes.

Real-World Outcomes	Estimates
**rwOS, overall**	
No. of pts	110
No. of events	85
Median (95% CI), months	18.9 (13.8–28.1)
1-year rate	64.5% (56.2–74.1)
2-year rate	41.8% (33.5–52.1)
3-year rate	28.0% (20.6–38.0)
5-year rate	15.4% (8.7–27.4)
**rwOS, palliative systemic LoT1**	
No. of pts	90
No. of events	68
Median (95% CI), months	21.2 (16.1–31.8)
1-year rate	72.2% (63.5–82.1)
2-year rate	45.6% (36.3–57.1)
3-year rate	28.8% (20.7–40.2)
5-year rate	17.0% (9.2–31.4)
**rwPFS, palliative systemic LoT1**	
No. of pts	90
No. of events	78
Median (95% CI), months	10.9 (8.8–13.6)
1-year rate	44.4% (35.3–56.0)
2-year rate	23.1% (15.9–33.8)
3-year rate	13.0% (7.1–23.9)
5-year rate	NA
**Time to CNS metastasis, palliative systemic LoT1**	
No. of pts	90
No. of events	22
Median (95% CI), months	48.1 (36.0–NA)
1-year rate	87.6% (80.2–95.6)
2-year rate	79.3% (69.6–90.4)
3-year rate	65.7% (53.0–81.5)
5-year rate	44.7% (28.3–70.5)
**rwTTNT (LoT1 to LoT2), palliative systemic LoT1**	
No. of pts	90
No. of events	42
Median (95% CI), months	23.9 (15.7–33.8)
1-year rate	71.6% (61.9–82.8)
2-year rate	48.3% (37.2–62.8)
3-year rate	26.7% (15.6–45.7)
5-year rate	NA
**rwToT, palliative systemic LoT1**	
No. of pts	90
No. of events	67
Median (95% CI), months	17.8 (13.1–25.3)
1-year rate	63.3% (54.1–74.1)
2-year rate	39.9% (31.0–51.5)
3-year rate	30.1% (21.8–41.5)
5-year rate	19.7% (11.7–33.0)

**Table 4:** Real-world clinical outcomes stratified by ECOG and mutation type.

rwOS, Overall	ECOG 0–1	ECOG 2–4	*p*-Value
No. of pts	73	37	0.008
No. of events	54	31	
Median (95% CI), months	22.8 (19.3–32.7)	10.3 (5.8–18.5)	
1-year rate	74.0 (64.6–84.8)	45.9 (32.4–65.2)	
2-year rate	49.3 (39.1–62.2)	27.0 (15.9–45.9)	
3-year rate	33.2 (23.8–46.2)	18.0 (8.9–36.4)	
5-year rate	18.7 (10.4–33.8)	NA	
**rwOS, overall**	**E 19 del**	**L858R**	***p*** **-value**
No. of pts	64	35	0.048
No. of events	47	29	
Median (95% CI), months	24.2 (19.3–33.1)	15.8 (10.3–27.3)	
1-year rate	70.3 (60.0–82.4)	62.9 (48.7–81.1)	
2-year rate	50.0 (39.1–63.9)	31.4 (19.3–51.3)	
3-year rate	33.4 (23.5–47.5)	22.5 (12.1–41.9)	
5-year rate	20.9 (11.8–37.2)	NA	
**rwOS, pts who started palliative systemic LoT1**	**ECOG 0–1**	**ECOG 2–4**	***p*** **-value**
No. of pts	62	28	0.086
No. of events	46	22	
Median (95% CI), months	26.4 (19.4–32.8)	12.9 (10.1–31.8)	
1-year rate	79.0 (69.5–89.8)	57.1 (41.5–78.8)	
2-year rate	51.6 (40.6–65.7)	32.1 (18.8–55.1)	
3-year rate	32.9 (22.9–47.3)	20.1 (9.4–43.0)	
5-year rate	18.6 (9.7–35.6)	NA	
**rwOS, pts who started palliative systemic LoT1**	**E 19 del**	**L858R**	***p*** **-value**
No. of pts	55	27	0.066
No. of events	39	22	
Median (95% CI), months	28.8 (21.0–38.0)	16.2 (13.6–28.1)	
1-year rate	76.4 (65.9–88.5)	74.1 (59.3–92.6)	
2-year rate	54.5 (42.9–69.4)	33.3 (19.6–56.8)	
3-year rate	35.7 (25–51.1)	21.6 (10.4–44.9)	
5-year rate	23.9 (13.6–42.1)	NA	
**rwPFS, pts who started palliative systemic LoT1**	**ECOG 0–1**	**ECOG 2–4**	***p*** **-value**
No. of pts	62	28	0.640
No. of events	54	24	
Median (95% CI), months	12.0 (9.8–19.2)	8.9 (7.1–20.7)	
1-year rate	48.4 (37.4–62.6)	35.7 (21.7–58.7)	
2-year rate	25.5 (16.6–39.1)	17.9 (8.1–39.5)	
3-year rate	13.6 (6.7–27.6)	11.9 (3.9–36.8)	
5-year rate	NA	NA	
**rwPFS, pts who started palliative systemic LoT1**	**E 19 del**	**L858R**	***p*** **-value**
No. of pts	55	27	0.100
No. of events	47	24	
Median (95% CI), months	12.2 (10.8–22.3)	9.0 (6.0–13.6)	
1-year rate	52.7 (41.1–67.7)	37.0 (22.6–60.6)	
2-year rate	29.1 (19.3–43.9)	14.8 (6.0–36.6)	
3-year rate	17.1 (9.3–31.5)	NA	
5-year rate	NA	NA	
**Time to CNS metastasis, pts who started palliative systemic LoT1**	**E 19 del**	**L858R**	***p*** **-value**
No. of pts	55	27	0.350
No. of events	13	6	
Median (95% CI), months	NA	36.0 (34.9-NA)	
1-year rate	91.6 (84.1–99.9)	84.9 (70.5–100)	
2-year rate	83.0 (72.1–95.6)	75.5 (56.1–100)	
3-year rate	73.3 (60.0–89.5)	50.3 (21.4–100)	
5-year rate	50.9 (31.4–82.4)	NA	
**rwTTNT (LoT1 to LoT2), pts who started palliative systemic LoT1**	**E 19 del**	**L858R**	***p*** **-value**
No. of pts	55	27	0.730
No. of events	27	12	
Median (95% CI), months	24.7 (14.1-NA)	23.2 (15.7-NA)	
1-year rate	70.7 (58.9–85.0)	74.4 (58.5–94.6)	
2-year rate	51.0 (38.0–68.4)	39.0 (20.6–73.7)	
3-year rate	29.9 (17.8–50.4)	NA	
5-year rate	NA	NA	
**rwToT (LoT1 to LoT2), pts who started palliative systemic LoT1**	**E 19 del**	**L858R**	***p*** **-value**
No. of pts	55	27	0.070
No. of events	38	22	
Median (95% CI), months	22.5 (18.3–37.3)	13.3 (10.5–25.3)	
1-year rate	70.9 (59.9–84.0)	59.3 (43.3–81.0)	
2-year rate	45.5 (34.0–60.7)	33.3 (19.6–56.8)	
3-year rate	37.7 (26.8–53.1)	20.7 (9.6–45.0)	
5-year rate	27.4 (17.2–43.7)	NA	

## Data Availability

The study dataset is not readily available due to privacy and ethical restrictions, but access may be requested from Portuguese Oncology Institute of Porto or Johnson & Johnson Innovative Medicine if reasonable and scientifically relevant.

## Supplementary Materials

The following supporting information can be downloaded at: https://www.mdpi.com/article/10.3390/curroncol32080414/s1, Figure S1: Other real-world outcomes by ECOG and mutation type; Figure S2: Real-world PFS by LoT1 TKI generation; Figure S3: Real-world OS by presence of brain metastasis at diagnosis; Table S1: Metastasis status at first disease progression, overall and by EGFR mutation category; Table S2: ECOG PS at disease progression timepoints, overall and by EGFR mutation category; Table S3: Weight at diagnosis and first progression, overall and by EGFR mutation category; Table S4: Real-world PFS for patients in systemic palliative treatment in LoT1 by TKI; Table S5: Real-world OS for patients in systemic palliative treatment in LoT1 by presence of brain metastasis at diagnosis.
