# Supplementary material for: Characterization of the Population, Treatment Patterns, and Outcomes of Patients with Advanced or Metastatic Non-Small-Cell Lung Cancer (NSCLC) with Epidermal Growth Factor Receptor Mutation (EGFRm): A Retrospective Cohort Study from IPO Porto
Source: Curr Oncol. 2025 Jul 24;32(8):414. doi: 10.3390/curroncol32080414 (PMC12384510; doi:10.3390/curroncol32080414)
Supplement: Supplementary file 1 [file curroncol-32-00414-s001.zip › curroncol-3713313-supplementary.pdf]

## SUPPLEMENTARY MATERIALS

### (a) Metastasis status at first disease progression

Metastasis status at first disease progression is described in Table S1.

**Table S1.** Metastasis status at first disease progression, overall and by EGFR mutation category

| | **Overall (*n* = 110)** | **Common EGFR mutation (*n* = 99)** | **Uncommon EGFR mutation (*n* = 11)** |
|---|---|---|---|
| Metastasis sites at 1st progression, *n* (%) | | | |
| 1 | 27 (24.5%) | 25 (25.3%) | ≤5 |
| 2 | 24 (21.8%) | 20 (20.2%) | ≤5 |
| 3 | 27 (24.5%) | 26 (26.3%) | ≤5 |
| 4 | 16 (14.5%) | 14 (14.1%) | ≤5 |
| 5 | 9 (8.2%) | Not shown | ≤5 |
| 6–7 | 7 (6.4%) | Not shown | ≤5 |
| Metastasis site group at 1st progression, *n* (%) | | | |
| 1–2 | 51 (46.4%) | 45 (45.5%) | 6 (54.5%) |
| 3–4 | 43 (39.1%) | Not shown | ≤5 |
| 5–7 | 16 (14.5%) | Not shown | ≤5 |
| Brain metastasis at 1st progression, *n* (%) | | | |
| No | 67 (60.9%) | 60 (60.6%) | 7 (63.6%) |
| Yes | 16 (14.5%) | Not shown | ≤5 |
| Metastasis at diagnosis and no progression | 14 (12.7%) | Not shown | ≤5 |
| Metastasis at diagnosis and progression | Not shown | 8 (8.1%) | 0 (0.0%) |
| Not applicable* | ≤5 | ≤5 | 0 (0.0%) |
| Liver metastasis at 1st progression, *n* (%) | | | |
| No | 82 (74.5%) | 74 (74.7%) | 8 (72.7%) |
| Yes | 6 (5.5%) | 6 (6.1%) | 0 (0.0%) |

*Patients in BSC only

### (b) ECOG PS at disease progression timepoints

Table S2 describes ECOG PS at disease progression timepoints.

**Table S2.** ECOG PS at disease progression timepoints, overall and by EGFR mutation category

| | **Overall (*n* = 110)** | **Common EGFR mutation (*n* = 99)** | **Uncommon EGFR mutation (*n* =11)** |
|---|---|---|---|
| ECOG PS at 1st progression | | | |
| 0–1 | 31 (28.2%) | 30 (30.3%) | ≤5 |
| 2 | 21 (19.1%) | 19 (19.2%) | ≤5 |
| 3 | 23 (20.9%) | 18 (18.2%) | ≤5 |
| 4 | 9 (8.2%) | 9 (9.1%) | 0 (0.0%) |
| Not applicable* | 11 (10.0%) | Not shown | ≤5 |
| On treatment (LoT1)† | 15 (13.6%) | Not shown | ≤5 |

| | | | |
|---|---|---|---|
| ECOG PS at 2nd progression | | | |
| 0 | 0 (0.0%) | 0 (0.0%) | 0 (0.0%) |
| 1 | 13 (11.8%) | Not shown | ≤5 |
| 2 | 7 (6.4%) | Not shown | ≤5 |
| 3–4 | 16 (14.5%) | 16 (16.1%) | 0 (0.0%) |
| Not applicable** | Not shown | 44 (44.4%) | 8 (72.7%) |
| On treatment (LoT1)† | 10 (9.1%) | Not shown | ≤5 |
| On treatment (LoT2)† | 9 (8.2%) | 9 (9.1%) | 0 (0.0%) |
| Missing | ≤5 | ≤5 | 0 (0.0%) |
| ECOG PS at 3rd progression | | | |
| 0 | 0 (0.0%) | 0 (0.0%) | 0 (0.0%) |
| 1 | ≤5 | ≤5 | ≤5 |
| 2 | ≤5 | ≤5 | 0 (0.0%) |
| 3 | ≤5 | ≤5 | 0 (0.0%) |
| 4 | ≤5 | ≤5 | 0 (0.0%) |
| Not applicable*** | 69 (62.7%) | 60 (60.6%) | 9 (81.8%) |
| On treatment (LoT1)† | 10 (9.1%) | Not shown | ≤5 |
| On treatment (LoT2)† | 9 (8.2%) | 9 (9.1%) | 0 (0.0%) |
| On treatment (LoT3)† | ≤5 | ≤5 | 0 (0.0%) |
| Missing | ≤5 | ≤5 | 0 (0.0%) |

*Patients in BSC only

**Patients did not initiate LoT2

***Patients did not initiate LoT3

†Patients did not progress and remain in the same LoT

**(c) Weight at diagnosis and at first progression**

Results regarding weight at diagnosis and at first progression are presented in Table S3.

**Table S3.** Weight at diagnosis and first progression, overall and by EGFR mutation category

| | **Overall (*n* = 110)** | **Common EGFR mutation (*n* = 99)** | **Uncommon EGFR mutation (*n* = 11)** |
|---|---|---|---|
| **Weight at diagnosis** | | | |
| Mean (SD) | 65.6 (12.2) | 64.6 (11.9) | 74.0 (11.6) |
| Median [Min, Max] | 64.0 [44.0, 101.0] | 63.0 [44.0, 101.0] | 73.0 [60.0, 98.5] |
| Unknown | ≤5 | ≤5 | 0 (0.0%) |
| **Weight at diagnosis** | | | |
| Less than 80 kg | 92 (83.6%) | 84 (84.8%) | 8 (72.7%) |
| 80 kg or more | Not shown | Not shown | ≤5 |
| Unknown | ≤5 | ≤5 | 0 (0.0%) |
| **Weight at 1st progression** | | | |
| Mean (SD) | 63.4 (12.2) | 62.2 (12.2) | 74.2 (4.87) |
| Median [Min, Max] | 64.5 [38.0, 90.0] | 63.0 [38.0, 90.0] | 74.2 [68.0, 79.0] |
| Not applicable | 11 (10.0%) | Not shown | ≤5 |
| On treatment (LoT1)† | 15 (13.6%) | Not shown | ≤5 |
| Unknown | 34 (30.9%) | Not shown | ≤5 |

| | | | |
|---|---|---|---|
| **Weight at 1st progression** | | | |
| Less than 80 kg | 46 (41.8%) | 41 (41.4%) | 5 (45.5%) |
| 80 kg or more | ≤5 | ≤5 | 0 (0.0%) |
| Not applicable* | Not shown | Not shown | ≤5 |
| On treatment (LoT1)† | 15 (13.6%) | Not shown | ≤5 |
| Unknown | 34 (30.9%) | Not shown | ≤5 |

*Not applicable—15 patients did not initiate any treatment.

†Patients did not progress and remained in the same LoT

**(d) Other analyses regarding real-world PFS, time to CNS metastasis, TTNT and rwToT**

Other comparisons for real-world PFS, time to CNS metastasis, TTNT and rwToT by ECOG and mutation are presented in Figure S1.

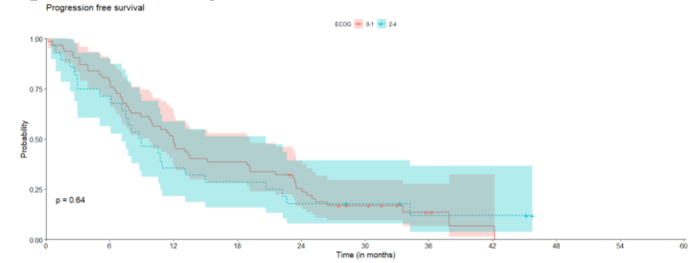


**(a)**

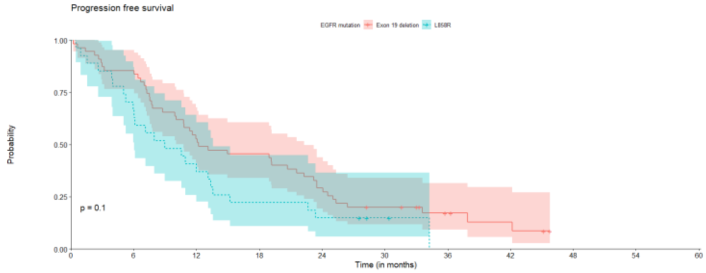


**(b)**

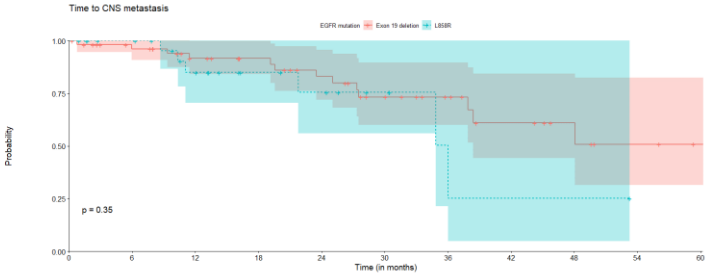


**(c)**

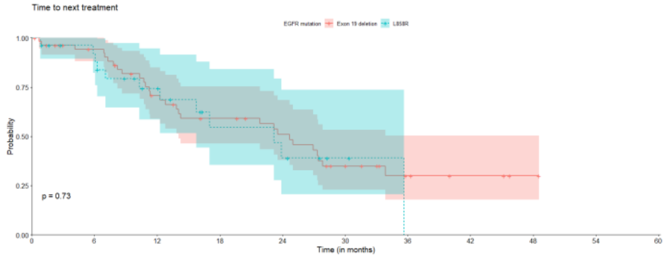


**(d)**

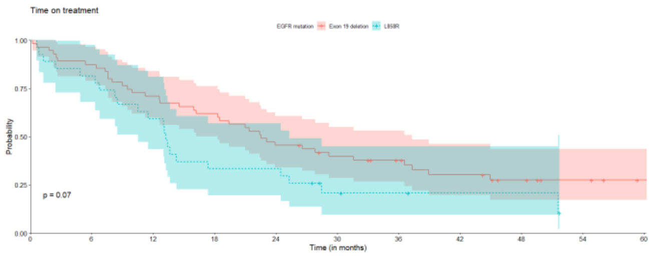


(e)

**Figure S1.** Other real-world outcomes by ECOG and mutation type. Real-world PFS (a) by ECOG (*n* = 90) and (b) by mutation (*n* = 82), (c) time to CNS metastasis by mutation (*n* = 82), (d) rwTTNT by mutation (*n* = 82), and (e) rwToT by mutation (*n* = 82) in the subgroup of patients who initiated LoT1 with systemic palliative treatment (*n* = 90).

**(e) Real-world PFS by TKI in LoT1**

The median rwPFS for patients who initiated palliative systemic treatment with TKI 1st generation in LoT1 was 10.7 months (95% CI, 8.0–13.6), 11.3 months (95% CI, 7.7–26.4) for TKI 2nd generation and 23.0 months for TKI 3rd generation (95% CI, 7.3–NA) ($p = 0.046$) (Table S4 and Figure S2).

**Table S4.** Real-world PFS for patients in systemic palliative treatment in LoT1 by TKI

| | **TKI 1st generation** | **TKI 2nd generation** | **TKI 3rd generation** | ***p*-value** |
|---|---|---|---|---|
| **rwPFS systemic palliative treatment** | | | | |
| No. of pts | 44 | 16 | 22 | 0.046 |
| No. of events | 41 | 16 | 13 | |
| Median (95% CI), months | 10.7 (8.0–13.6) | 11.3 (7.7–26.4) | 23.0 (7.3–NA) | |
| 1-year rate | 40.9 (28.7–58.4) | 43.8 (25.1–76.3) | 59.1 (41.7–83.7) | |
| 2-year rate | 13.6 (6.5–28.7) | 25.0 (10.7–58.4) | 45.0 (28.2–71.7) | |
| 3-year rate | 9.1 (3.6–23.1) | 6.2 (0.9–41.7) | NA | |
| 5-year rate | NA | NA | NA | |

NA: Not achieved

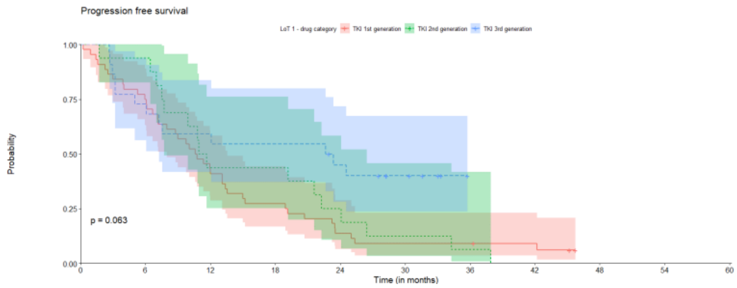


**Figure S2.** Real-world PFS by LoT1 TKI generation

**(f) Real-world OS from treatment initiation by presence of brain metastasis at diagnosis**

The median rwOS from treatment for patients who initiated palliative systemic treatment and without brain metastasis at diagnosis was 20.5 months (95% CI, 14.4–28.6) and for patients with brain metastasis at

diagnosis was 16.4 months (95% CI, 5.4–NA). No statistical difference was found between the curves ($p$ = 0.960) (Table S5 and Figure S3).

**Table S5.** Real-world OS for patients in systemic palliative treatment in LoT1 by presence of brain metastasis at diagnosis

| | **With brain metastasis** | **Without brain metastasis** | ***p*-value** |
|---|---|---|---|
| **rwOS systemic palliative treatment** | | | |
| No. of pts | 21 | 69 | 0.960 |
| No. of events | 15 | 53 | |
| Median (95% CI), months | 16.4 (5.4–NA) | 20.5 (14.4–28.6) | |
| 1-year rate | 68.1 (58.0–80.0) | 57.1 (39.5–82.8) | |
| 2-year rate | 43.4 (33.2–56.9) | 47.6 (30.4–74.6) | |
| 3-year rate | 27.6 (18.6–40.8) | 29.4 (14.2–60.8) | |
| 5-year rate | 17.3 (9.1–33.2) | NA (NA–NA) | |

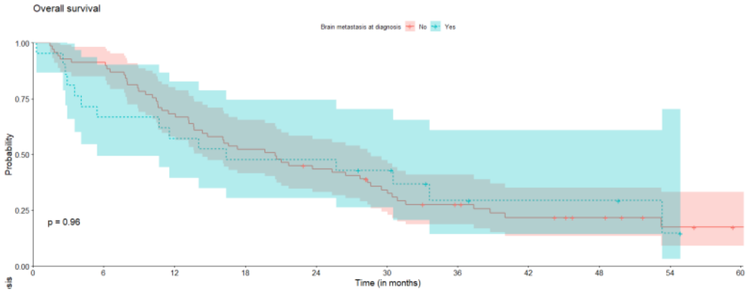


**Figure S3.** Real-world OS by presence of brain metastasis at diagnosis
